# Delivering an impactful presentation: a practical guide

**DOI:** 10.46989/001c.124436

**Published:** 2024-10-14

**Authors:** Mohamad Mohty

**Affiliations:** 1 Sorbonne Université

**Keywords:** presentations, audience engagement, body language, scientific communication

## Introduction

Presentations are a powerful and enduring form of communication in the scientific and medical fields. They offer an opportunity to engage directly with an audience, share key insights, and clarify complex data. Presentations allow for the dynamic delivery of content. Unlike static formats such as journal articles, they provide opportunities for real-time interaction. Speakers can emphasize key points, gauge the audience’s reactions, and address questions on the spot. However, the challenge lies in keeping the audience engaged from beginning to end, and despite their importance, many presentations fail to make an impact. Moreover, presentations come with significant costs, not only in terms of the time invested by the speaker but, more importantly, the time and attention of the audience. Consider an audience of 100 physicians, each dedicating valuable hours to attend a presentation, often flying across time zones and incurring great expense. The speaker’s responsibility is to ensure that this time investment results in meaningful engagement and knowledge transfer. This editorial aims to provide practical guidelines for delivering effective and memorable presentations, drawing on key principles of communication, storytelling, and audience engagement.

## Common pitfalls in presentations

Many presentations fail due to a lack of brevity, an overload of data, or a failure to craft a cohesive narrative. Successful presentations require more than just reciting methodology or reading from slides. The speaker must create a story, engage the audience, and use tools like body language and tone to enhance communication. Key reasons for failure include: (1) overloading with information: presenters often confuse the format of a presentation with that of a detailed academic paper, overwhelming the audience with excessive data; (2) lack of narrative: presentations without a clear storyline fail to captivate the audience. Speakers must engage listeners in a dialogue, creating a journey through the content; and (3) failure to engage: poor use of voice, body language, or ineffective movement can disconnect the audience from the speaker. The audience is indeed the true focus of any presentation. Understanding their needs, knowledge level, and expectations is critical. The speaker’s role is to tailor the presentation to suit the audience’s requirements, ensuring they remain engaged throughout.

## Structuring a presentation

To hold the audience’s attention, a presentation must be structured like a well-told story, and as such, must have a clear beginning, an engaging narrative, and a strong conclusion. The speaker should craft a natural flow, dividing the talk into distinct sections, and use hooks to make the content memorable. The opening words are crucial in setting the tone. Whether through a rhetorical question, a provocative statement, an unusual fact, or a humorous anecdote, the speaker must grab the audience’s attention immediately. The final words are just as important as the opening. A strong finish leaves a lasting impression. The conclusion should be short, direct, and delivered with conviction, ensuring the audience takes away the key message. Visual aids such as graphs, tables, and animations are essential for illustrating complex data, but they must be chosen carefully. Overly complicated visuals can detract from the message. Each piece of supporting material should be clearly labeled, well-referenced, and directly linked to real-world outcomes. The presenter must control tools like laser pointers effectively, ensuring they enhance, rather than distract from, the message.

## Conveying the content

The speaker’s choice of words and delivery style is critical. Keeping the message simple and short is a guiding principle. The speaker should avoid unnecessary jargon or overly technical language, opting instead for clarity and brevity. Additionally, the rhythm, speed, volume, and use of pauses play a significant role in maintaining the audience’s attention: (1) the speaker’s voice should have variation and movement, like a musical instrument (rhythm). A monotonous tone will result in the audience losing interest; (2) depending on the audience’s background, the speaker may need to slow down to ensure comprehension, particularly if the audience includes non-native speakers (speed); (3) projection is crucial. The speaker should practice speaking to the back of the room, ensuring everyone can hear clearly (volume); and (4) strategic pauses allow the audience time to absorb key points (pauses).

**Figure 1.**
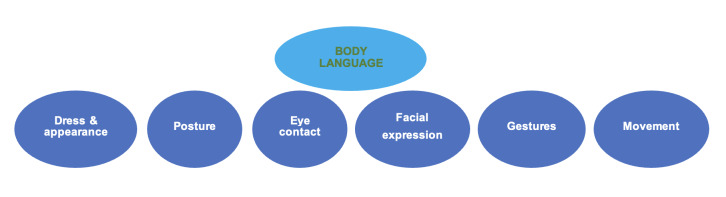
Components of the body language to exhibit

Effective body language is another fundamental aspect of communication. The audience must be able to see the speaker clearly at all times, and the speaker should use movement, posture, facial expressions, and gestures to reinforce the message. Confidence in body language is key to building rapport with the audience and enhancing the overall delivery (**Figure 1**).

Finally, it is natural to feel nervous before a presentation. Speakers should embrace this energy and channel it into enthusiasm. Techniques such as deep breathing, arriving early to check the setup, and mingling with the audience beforehand can help alleviate anxiety. Rehearsing the presentation out loud, with gestures and facial expressions, is essential for building confidence.

## Concluding remarks

Delivering an impactful presentation requires careful planning, storytelling, and audience engagement. By focusing on the audience’s needs, structuring the talk as a narrative, and using well-chosen visuals and body language, speakers can ensure their presentations are not only informative but memorable. Ultimately, the goal is to leave the audience with a clear, concise message that resonates long after the presentation is over.

### AUTHORSHIP

Writing, editing, conceptualization: Mohamad Mohty (Lead).

### STATEMENTS AND DECLARATIONS

Conceptualization: Mohamad Mohty (Lead). Writing – original draft: Mohamad Mohty (Lead). Writing – review & editing: Mohamad Mohty (Lead).

### ETHICAL APPROVAL

Not applicable.

### CONSENT TO PARTICIPATE/INFORMED CONSENT

Not applicable.

### CONSENT FOR PUBLICATION

Not applicable.

